# The purine receptor P2X7R regulates the release of pro-inflammatory cytokines in human craniopharyngioma

**DOI:** 10.1530/ERC-16-0338

**Published:** 2017-04-07

**Authors:** Jing Nie, Guang-long Huang, Sheng-Ze Deng, Yun Bao, Ya-Wei Liu, Zhan-Peng Feng, Chao-Hu Wang, Ming Chen, Song-Tao Qi, Jun Pan

**Affiliations:** 1 Department of NeurosurgeryNanfang Hospital, Southern Medical University, Guangzhou, China; 2 Nanfang Neurosurgery Research InstitutionNanfang hospital, Southern Medical University, Guangzhou, China

**Keywords:** craniopharyngioma, pituitary, P2X7 receptor, cytokine

## Abstract

Craniopharyngiomas (CPs) are usually benign, non-metastasizing embryonic malformations originating from the sellar area. They are, however, locally invasive and generate adherent interfaces with the surrounding brain parenchyma. Previous studies have shown the tumor microenvironment is characterized by a local abundance of adenosine triphosphate (ATP), infiltration of leukocytes and elevated levels of pro-inflammatory cytokines that are thought to be responsible, at least in part, for the local invasion. Here, we examine whether ATP, via the P2X7R, participates in the regulation of cytokine expression in CPs. The expression of P2X7R and pro-inflammatory cytokines were measured at the RNA and protein levels both in tumor samples and in primary cultured tumor cells. Furthermore, cytokine modulation was measured after manipulating P2X7R in cultured tumor cells by siRNA-mediated knockdown, as well as pharmacologically by using selective agonists and antagonists. The following results were observed. A number of cytokines, in particular IL-6, IL-8 and MCP-1, were elevated in patient plasma, tumor tissue and cultured tumor cells. P2X7R was expressed in tumor tissue as well as in cultured tumor cells. RNA expression as measured in 48 resected tumors was positively correlated with the RNA levels of IL-6, IL-8 and MCP-1 in tumors. Furthermore, knockdown of P2X7R in primary tumor cultures reduced, and stimulation of P2XR7 by a specific agonist enhanced the expression of these cytokines. This latter stimulation involved a Ca^2+^-dependent mechanism and could be counteracted by the addition of an antagonist. In conclusion, the results suggest that P2X7R may promote IL-6, IL-8 and MCP-1 production and secretion and contribute to the invasion and adhesion of CPs to the surrounding tissue.

## Introduction

Neoplasms that grow slowly and lack the ability to form metastases are usually termed benign. Some of them, however, are locally invasive and damage the surrounding tissue. A point in case are craniopharyngiomas (CPs), which are suprasellar tumors derived from the pituitary (Garnett *et al*. 2007, Puget *et al*. 2007, Karavitaki & Wass 2008, Larkin & Ansorge 2013). They are characterized by indistinct margins, adherence to neighboring brain tissue and association with reactive gliosis, all of which render a distinction between tumor and healthy tissue difficult during surgical resection. In fact, morbidity and mortality associated with these tumors are usually high (Garnett *et al*. 2007, Muller 2011, 2013). Radical resection may severely damage critical local structures (Banna 1976, Miller 1994), including the pituitary stalk, cranial and optic nerves and the hypothalamus. However, conservative resection is frequently associated with tumor recurrence. Interestingly, however, the same anatomical region may also give rise to other tumors, such as pituitary adenoma, which are rarely invasive, and the prognosis is much better than that of CPs. It is, therefore, of paramount importance to determine the pathogenetic mechanisms that control local tumor invasion.

It is well established that elevated inflammatory responses are associated with poor outcomes in neoplastic disease (Neuman 2007, Roxburgh & McMillan 2014). It is thought that these inflammatory responses may favor a tumor-promoting microenvironment by increasing angiogenesis and helping tumor cells escape immune surveillance (Wang & DuBois 2015). In our own research, we have previously found that brain tissue adjacent to adamantinomatous CP (aCP) is infiltrated by leukocytes and marked by the presence of cytokines (Zhou *et al*. 2013). These cytokines, which may be produced by both leukocytes and tumor cells themselves, may generate a microenvironment that is permissive to CP invasion (Balkwill & Coussens 2004). IL-6, for example, may play a major role in tumorigenesis (Grivennikov & Karin 2008, Chalaris *et al*. 2011) and be involved in the inflammatory process at the interface between CP and brain parenchyma (Lister *et al*. 2007). IL-8 has been shown to be involved in glioma cell proliferation and angiogenesis (de la Iglesia *et al*. 2008), and MCP-1 increases macrophage recruitment, angiogenesis, invasion and metastasis (Conti & Rollins 2004). Hence, here, we investigated which specific cytokines are elevated in CP and which mechanisms might lead to their elevation. We found that among ten cytokines with elevated expression, IL-6, IL-8 and MCP-1 are particularly highly expressed in CP. An earlier study showing that large amounts of ATP in the hundreds micromolar range can be found in the microenvironment of transplanted tumors in mice (Pellegatti *et al*. 2008). ATP activates P2 receptors on plasma membranes, and their activation regulates many physiological functions (Burnstock 2009). P2 receptors are divided into two subfamilies, metabotropic P2Y1-14 receptors and ionotropic P2X1-7 receptors. Among the latter, the ligand-gated ion channel, P2X7R, whose expression is ubiquitous in most tissues (Burnstock & Knight 2004) and elevated in many types of cancers (Greig *et al*. 2003, Slater *et al*. 2004, Raffaghello *et al*. 2006, Solini *et al*. 2008, Adinolfi *et al*. 2012, Takai *et al*. 2014), is of particular interest here as its activation is associated with cytokine release in both immune and non-immune cells (Solini *et al*. 2000, Inoue *et al*. 2007, Lister *et al*. 2007, Fang *et al*. 2011, Friedle *et al*. 2011, Hung *et al*. 2013, Killeen *et al*. 2013, Xu *et al*. 2013, Bianchi *et al*. 2014, Shieh *et al*. 2014, Braganhol *et al*. 2015, Sathanoori *et al*. 2015), and hence, with the modulation of inflammatory responses. We find that P2X7R is indeed expressed in CP and that its activation leads to the stimulation of cytokine release in CP-derived cultured cells in a Ca^2+^-dependent manner.

## Materials and methods

### Subjects

Plasma and surgical samples from 48 patients with a postoperative pathological diagnosis of CP were studied. They were selected based on a retrospective review of the medical records of patients visiting the Neurosurgery Department of Nanfang Hospital, Southern Medical University between January 2013 and August 2015. The patients’ ages ranged from 3 to 68 years. Twenty demographically matched healthy volunteers were enrolled as donors of control plasma. Subjects were excluded if they had a prior history of significant neurological disorder, head trauma, mental retardation or recent substance abuse. The study was approved by the Ethics Committee (Institutional Review Board) of the Southern Medical University, and patient records were anonymized prior to analysis.

### Materials

2′(3′)-O-(4-Benzoylbenzoyl) adenosine 5′-triphosphate (BzATP), adenosine 5′-triphosphate-2′,3′-dialdehyde (oxATP) and Kn62 were purchased from Sigma Chemical Co. (St. Louis, USA). The P2X7R antibody was from Alomone Labs (Jerusalem, Israel, Cat: APR-004). The pan-cytokeratin (pan-CK), vimentin, GAPDH and HRP-coupled goat anti-rabbit IgG antibodies were from Abclonal Technology. Alexa Fluor 555-coupled secondary antibody and DAPI were from Life Technologies, and Lipofectamine 2000 was from Invitrogen. Cytokine levels (IFNα2, IFN-γ, IL-10, IL-1α, IL-1β, IL-6, IL-8, MCP-1, MIP-1α and TNF-α) were measured using Millipore bead arrays (Milliplex MAP multiplex kit, Millipore, Billerica, MA, USA).

### Cell culture

Primary human CP cells were prepared and cultured from tumor samples after intraoperative diagnosis had been confirmed on frozen sections by an experienced pathologist. The tumor samples were immediately placed into Dulbecco’s phosphate-buffered saline (without calcium and magnesium, pH = 7.2) and stored at 4°C. Tumor specimens were cut into small pieces and washed in Dulbecco’s phosphate-buffered saline solution containing 1% penicillin/streptomycin, and incubated with trypsin (0.25%) for 30 min at 37°C and 5% CO_2_. The reaction was stopped by adding 10% fetal bovine serum (Gibco) in Dulbecco’s Modified Eagle Medium (DMEM, Gibco). The cells were centrifuged at 1000 rpm for 5 min at room temperature, gently re-suspended in 4 mL DMEM containing 10% fetal bovine serum, 100 U/mL penicillin and 100 mg/mL streptomycin sulfate, and cultured in 25 mL bottles at 37°C in a humidified atmosphere with 5% CO_2_. The medium was changed every 2–3 days. Positive identification of the cells was achieved by positive staining for pan-cytokeratin and absence of staining for vimentin (Li *et al*. 2013).

### Measurement of cytokine expression in plasma, tissue and cultured cells

Plasma was obtained from peripheral blood in EDTA and immediately stored in liquid nitrogen. Cytokines were measured using Millipore bead arrays according to the manufacturer’s guidelines. CP tissues were washed with 2 mL of sterile 1× phosphate-buffered saline (PBS), collected by centrifugation and stored in liquid nitrogen for further analysis. Primary cultured CP cells were trypsinized, centrifuged at 3000 rpm and washed twice with cold PBS. Tissues and cells were incubated in lysis buffer (1% NP-40, 1% Triton X-100, 0.1% SDS) and centrifuged at 10,000 rpm for 10 min. Total protein concentrations were measured using a Bio-Rad DC kit (Bio-Rad) and 30 µg of protein were analyzed using the same Millipore bead arrays as for plasma. In addition, cultured cells were used for quantitative RT-PCR.

### Immunohistochemistry of tissue sections

Immunohistochemistry was carried out on 4 µm sections of paraffin-embedded tissues. After deparaffinization and rehydration, the sections were heated for 20 min in sodium citrate buffer (pH 6.0) using a microwave oven. Endogenous peroxidase was blocked by incubating in 3% hydrogen peroxide for 10 min. The sections were incubated overnight at 4°C with rabbit polyclonal anti-P2X7R (dilution 1:300), washed, incubated with HRP-coupled goat anti-rabbit IgG and staining was visualized using 3,3′-diaminobenzidine (DAB) chromogen for 1 min. The sections were counterstained using hematoxylin, dehydrated and then mounted using permount. For negative controls, the primary antibody was replaced with PBS.

### Immunofluorescence of cultured cells

Primary cultured CP cells were seeded on glass cover slips, rinsed with phosphate-buffered saline (PBS) and fixed with paraformaldehyde (4% in PBS) for 30 min. The cells were permeabilized with Triton X-100 (0.5% in PBS) and incubated for 20 min in FBS (2% in PBS), rinsed and incubated at 4°C overnight with the rabbit polyclonal anti-P2X7R (dilution 1:100). Cells were then rinsed three times with PBS and incubated for 30 min with an anti-rabbit Ig FITC-labeled antibody (Alexa Fluor 555 secondary antibody:dilution 1:1000). At the end of this incubation, cover slips were rinsed three times. Finally, the cells were counterstained with 4′,6-diamidino-2-phenylindole dihydrochloride (DAPI) for 5 min. Fixed cells stained with anti-rat IgG antibodies served as negative controls. All staining procedures were performed at room temperature. Representative fluorescence photographs were taken using a spectral confocal microscope (Olympus). The photographs were analyzed by ZEN 2009 light edition software (Zeiss).

### siRNA transfection

siRNAs targeting specific sequences of P2X7R and a negative control (scrambled siRNA, no homology to any human gene sequence) were synthesized by Gene Pharma Co. Ltd. (Shanghai, China). The optimal P2X7R-siRNA was selected based on the results of real-time PCR and Western blotting. The siRNA sequences directed against P2X7R were sense: 5′-GGAUCCAGAGCAUGAAUUAUU-3′, anti-sense: 5′-UAAUUCAUGCUCUGGAUCCUU-3′. Transfections of control and P2X7R-siRNA were performed using Lipofectamine 2000 (Invitrogen) according to the manufacturer’s instructions.

### Quantitative real-time PCR

Total RNA was isolated from CP cells using the TRIzol total RNA reagent (TaKaRa Biotechnology Co.). cDNA synthesis was performed with 2 µg total RNA using the PrimeScript RT Master Mix Reagent Kit (RR 036A Takara-Bio, Otsu, Japan). The primers were designed with Primer Express 3.0 software, and the sequences were as follows: IL-6, forward, 5′-AAAGAGGCACTGGCAGAAAA-3′; reverse, 5′-TTTCACCAGGCAAGTCTCCT-3′; IL-8, forward, 5′-AAGAAACCACCGGAAGGAAC-3′; reverse, 5′-ACTCCTTGGCAAAACTGCAC-3′; MCP-1/CCL-2, forward, 5′-AAGAAACCACCGGAAGGAAC-3′; reverse, 5′-ACTCCTTGGCAAAACTGCAC-3′; P2X7R, forward, 5′-ACAGGAAGAAGTGCGAGTCC-3′; reverse, 5′-GGTAGAGCAGGAGGAACTGC-3′; GAPDH, forward, 5′-GAAGGTGAAGGTCGGAGTC-3′; reverse, 5′-GAAGATGGTGATGGGATTTC-3′. Quantitative PCR was performed using the Light Cycler 480 Real-time PCR System (Roche). The quantification of gene expression was performed using the ▵▵CT calculation with CT as the threshold cycle. The relative levels of target genes, normalized to the sample with the lowest CT, are given as 2^−▵▵Ct^ (Livak & Schmittgen 2001).

### Western blot

Primary cultured CP cells, treated under different conditions, were minced and placed into pre-chilled RIPA buffer (50 mM Tris–HCl, pH 7.4, 150 mM NaCl, 1% Triton X-100, 1% sodium deoxycholate, 0.1% SDS, 1 mM EDTA, PMSF 10 mL/mL and 1× protease inhibitor cocktail (Sigma Aldrich)). Samples were then homogenized and cleared by centrifugation at 10,000 ***g*** for 10 min. Total protein content was measured using a BCA (bicinchoninic acid assay) protein assay reagent kit. Lysates were mixed with Laemmli buffer and boiled for 5 min. 30 µg of protein were separated on a 12% SDS-PAGE gel followed by transfer to polyvinylidene difluoride (PVDF) membrane (Millipore), after which membranes were blocked with 5% non-fat dry milk in PBS/0.1% Tween20 (PBST) solution. Then, the membranes were incubated with anti-P2X7R (1:1000) in TBS-T containing 1% bovine serum albumin (BSA) overnight. After washing with TBS-T, the membranes were incubated with HRP goat anti-rabbit IgG polyclonal antibody (1:1000 in TBS-T containing 1% BSA, Abclonal Technology) for 1 h at room temperature and then washed three times. Chemiluminescence reactions were carried out with ECL substrate (Millipore), exposed to film according to the manufacturer’s directions and analyzed using Fusion image acquisition system (Peqlab, Erlangen, Germany). To confirm protein loading, membranes were probed with antibody recognizing GAPDH (1:1000). The Western blot data shown are representative for at least three independent experiments.

### Data analysis and statistics

All experiments were performed in triplicate, and the data are presented as the means ± s.e.m. of at least 3 independent experiments. The differences between the sample means were compared using analysis of variance. All analyses were performed using SPSS for Windows, version 11.5 (SPSS), using an unpaired *t*-test, one-way ANOVA analysis with *post hoc* Tukey’s test or Spearman’s rank correlation coefficient followed by simple main effect test. *P* < 0.05 was considered statistically significant.

## Results

### Pro-inflammatory cytokine levels are increased in CP

**Figure 1:**
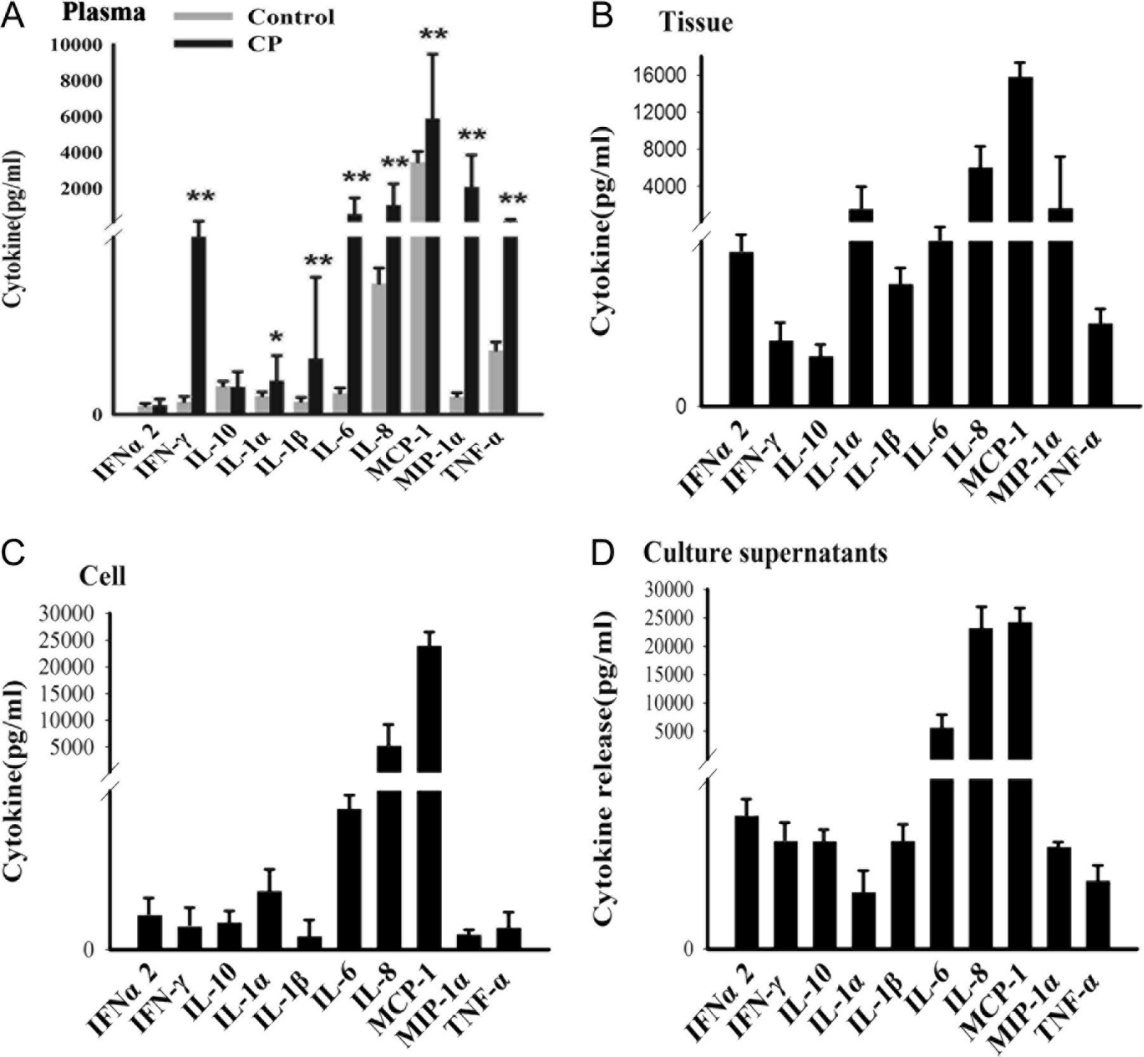
Cytokine levels associated with CP. Cytokine levels in plasma, CP tissue or cells and their supernatants were measured using Millipore bead arrays. (A) Comparison of plasma cytokine levels in CP patients collected before operation and demographically matched healthy controls. (B) Expression in CP tissues. (C) Expression in primary cultured CP cells. (D) Expression in supernatants of primary cultured CP cells. Note particularly high levels of expression of IL-6, IL-8 and MCP-1 in all samples. *P < 0.05 vs. healthy control group; **P < 0.01 vs. healthy control group.

We first tested whether the increased levels of pro-inflammatory cytokines around CP tissue observed previously (Zhou *et al*. 2013) would be associated with increased plasma levels of cytokines. As shown in Fig. 1A, of the 10 cytokines tested, IFN-γ, IL-1α, IL-1β, IL-6, IL-8, MCP-1, MIP-1α and TNF-α were all significantly upregulated in the plasma of CP patients compared to plasma from healthy controls (*P* < 0.05). Among these, IL-6, IL-8, MCP-1 and MIP-1α reached particularly high levels, and IL-6, IL-8 and MCP-1 were consistently among the cytokines with the highest expression levels in CP tissue, cultured CP cells and the supernatant of these cultured cells (Fig. 1B–D). These results suggested that pro-inflammatory cytokines are elevated locally around the tumor and in plasma and that tumor cells themselves may be a source for these elevated levels.

### Expression of purinergic P2X7R in CP

**Figure 2:**
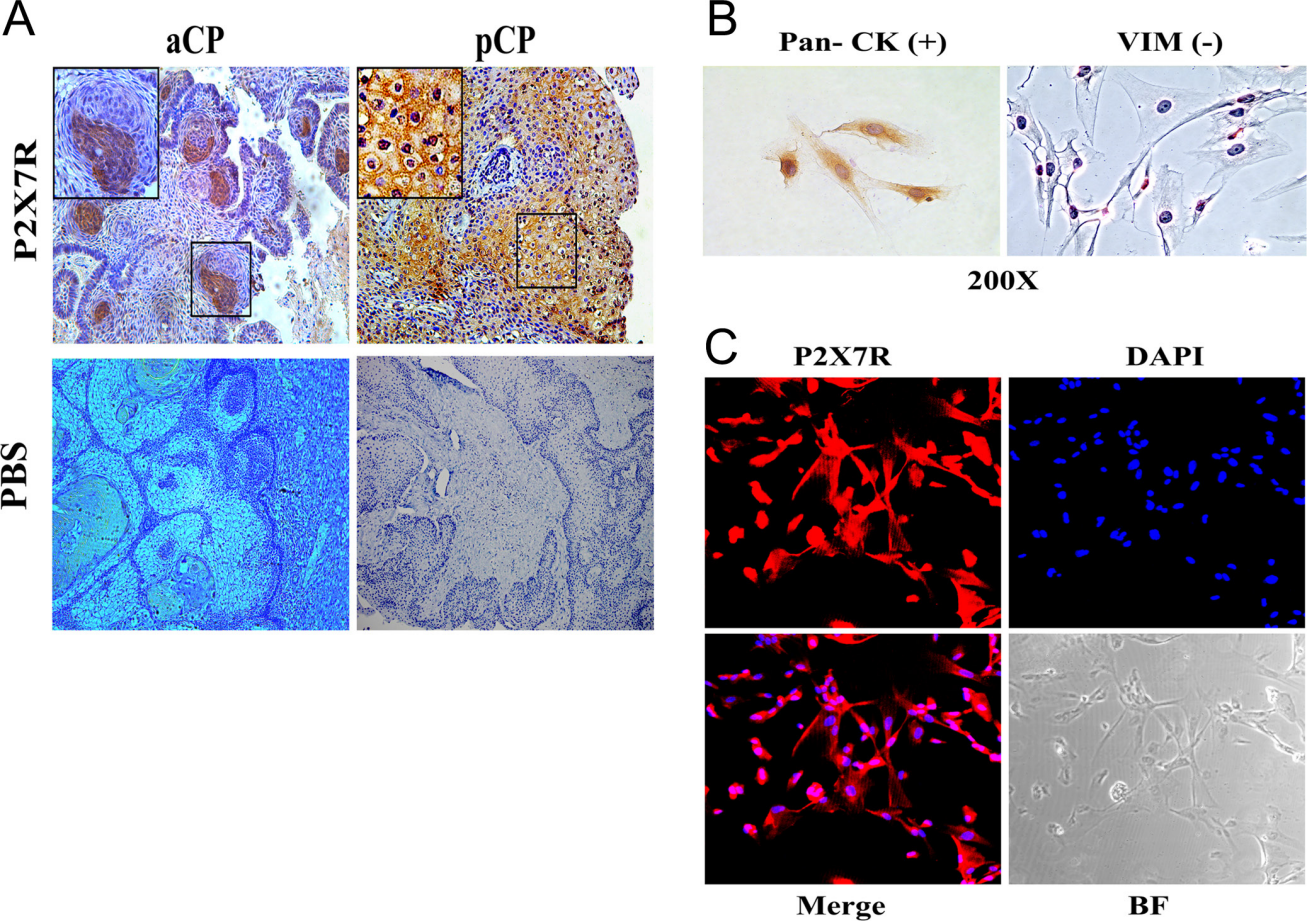
Expression of the purinergic P2X7R in vivo and in cultured cells. immunohistochenistry of a tissue section of an adamantinomatous CP tumor (aCP, A) and a papillary CP tumor (pCP, B). Note labeling in whirl-like cellular structures in aCP and homogeneous staining in pCP. (C) Identification of primary aCP cells in culture. Tumor cells are characterized by positive staining for pan cytokeratin (pan CK, left panel) and absence of staining for vimentin (VIM, right panel). (D) Immunofluorescence for P2X7R along with DAPI labeling, merged panel and bright field (BF) microscopy as indicated. Note positive staining for P2X7R in tumor cells.

The suggestion that locally elevated ATP levels may participate in cytokine upregulation through activation of P2X7R then prompted us to determine whether this ion channel is indeed expressed in CP tissue *in vivo* and in cultured CP cells *in vitro*. As shown in Fig. 2A and B, immunohistochemistry on sections of resected CP tumors clearly showed P2X7R staining. This staining was not only seen in craniopharyngiomas of the adamantinomatous type (aCP), where it appeared in whirl-like cell clusters (aCP, Fig. 2A), but also in those of the papillary type (pCP) which are only seen in adults and which exhibited staining in a homogeneous membraneous pattern (Fig. 2B). We then confirmed the expression within the tumors by directly labeling cultured tumor cells obtained from ten different aCP specimens. These cells were identified as tumor cells by positive staining for pan-cytokeratin and absence of staining for vimentin (Fig. 2C). As shown in Fig. 2D, such tumor cells show positive staining for P2X7R. Hence, P2X7R is expressed both in CP *in vivo* and their tumor cells *in vitro*.

### Correlation between P2X7R and cytokine expression in CP patients

**Figure 3:**
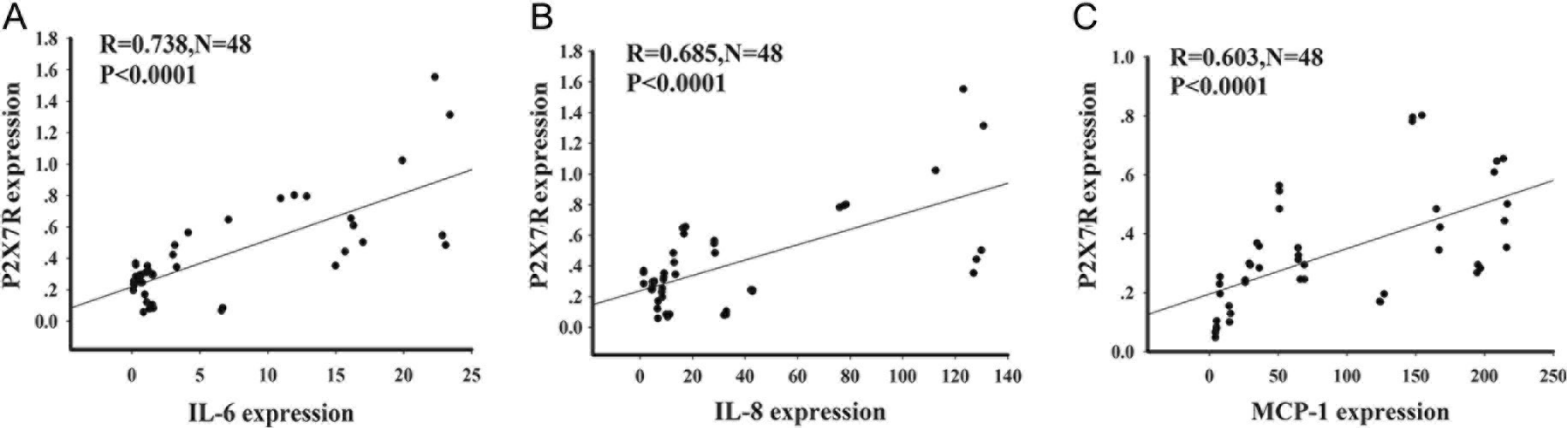
Correlation between the relative RNA expression levels of P2X7R and IL-6(A), IL-8 (B), and MCP-1(C) in 48 individual CP tissues is shown. Spearman’s rank correlation coefficient (R) was calculated. N: number of samples. *P* < 0.05 was considered significant.

To evaluate whether P2X7R expression was positively correlated with elevated expression of IL-6, IL-8 and MCP-1, we determined the expression of the respective RNAs by real-time PCR in tumor tissues from all 48 patients and correlated the expression levels using Spearman’s rank correlation coefficient. As shown in Fig. 3, this analysis revealed, as expected, a wide variation in expression levels of P2X7R and cytokine RNAs among the individual patient samples. The RNA levels of P2X7R and the individual cytokines, however, were positively correlated, with the respective *R* values being 0.738 for IL-6, 0.658 for IL-8, and 0.607 for MCP-1, all at *P* < 0.0001.

### RNA expression and cytokine release in response to P2X7R activation

**Figure 4:**
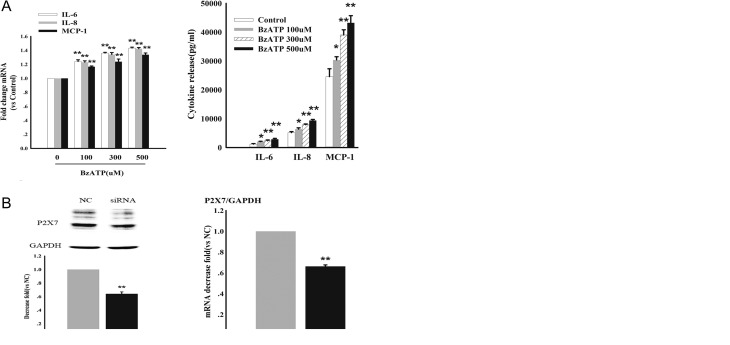
P2X7R modulates IL-6, IL-8 and MCP-1 expression and release. Primary cultured aCP cells were incubated with different concentrations of the P2X7R agonist BzATP for 48 hours. Levels of cytokine RNAs were analyzed by real time RT-PCR (A, left panel) and cytokine release was analyzed by Millipore multiplex bead array (A, right panel). (B) Effect of transfection with P2X7R siRNA and control siRNA on primary cultured CP cells. Fourty-eight hours after transfection, cells were analyzed by western blot (B, left upper panels for gel electrophoresis, left lower panel for quantification) and by RT-PCR normalized to the levels of GAPDH (B, right panel). Note reduction of approximately 40% of both P2X7R RNA and protein in presence of specific siRNA. (C, left panel) Relative RNA levels (fold change after P2X7R siRNA compared to control siRNA) of IL-6/IL-8 and MCP-1 as determined by RT-PCR. (C, right panel) corresponding cytokine release. (D) P2X7R antagonist oATP or Kn62 significantly attenuates BzATP induced IL-6/IL-8 and MCP-1 expression. Cells were pretreated with vehicle (250 mM Hepes) or the P2X7R antagonists oATP (300 mM) or Kn62 for 2 h, prior to stimulation with either vehicle or BzATP (300 mM) for 48 hours. The change of mRNA and release levels were analyzed by real time RT-PCR (D left) and Millipore multiplex bead array (D right). Data are representative of three independent experiments; bars represent the means ± s.e.m. from triplicates. Statistical significance was determined by ANOVA analysis with post hoc Tukey’s test. *P < 0.05 vs. respective control Group; **P < 0.01 vs. respective control Group; ^#^*P* < 0.05 vs. the cultures exposed to BzATP (300 mM) alone; ^##^*P* < 0.01 vs. the cultures exposed to BzATP (300 mM) alone.

To test whether activation of P2X7R might be functionally involved in the above-suggested elevation of cytokine levels, we first activated the ion channel in cultured CP cells using the potent non-hydrolysable benzoyl derivative of ATP (BzATP) as a P2X7R agonist. As shown in Fig. 4A , 48 h after exposure of cells to BzATP, there was a dose-dependent increase in both RNA expression and release of IL-6, IL-8 and MCP-1. To confirm that P2X7R was involved in this reaction, we then transfected the cells with P2X7R-specific siRNAs to knock down P2X7R expression. As shown in the Western blots in Fig. 4B, siRNA reduced P2X7R expression by around 40%, concomitant with the reduction in the corresponding P2X7R RNA. This reduction was correlated with a similar reduction in the RNA levels of IL-6, IL-8 and MCP-1 and the release of the corresponding cytokines. These results suggested that even in the absence of extraneous activation of P2X7R, its levels regulate cytokine expression and secretion. To confirm this observation, we then treated cells with the P2X7R antagonist periodate-oxidized ATP (oATP, 300 µM) or Kn62 (2.5 µM) for 2 h, followed by treatment with vehicle or BzATP for 48 h. As shown in Fig. 4D, although oATP or Kn62 alone did not significantly change the levels of cytokine RNAs or cytokine release, it was able to reduce the effect of BzATP. Hence, taken together, P2X7R appears to be causally involved in the regulation of the respective cytokines.

### P2X7R activation stimulates IL-6, IL-8 and MCP-1 expression through a Ca^2+^-dependent mechanism

**Figure 5:**
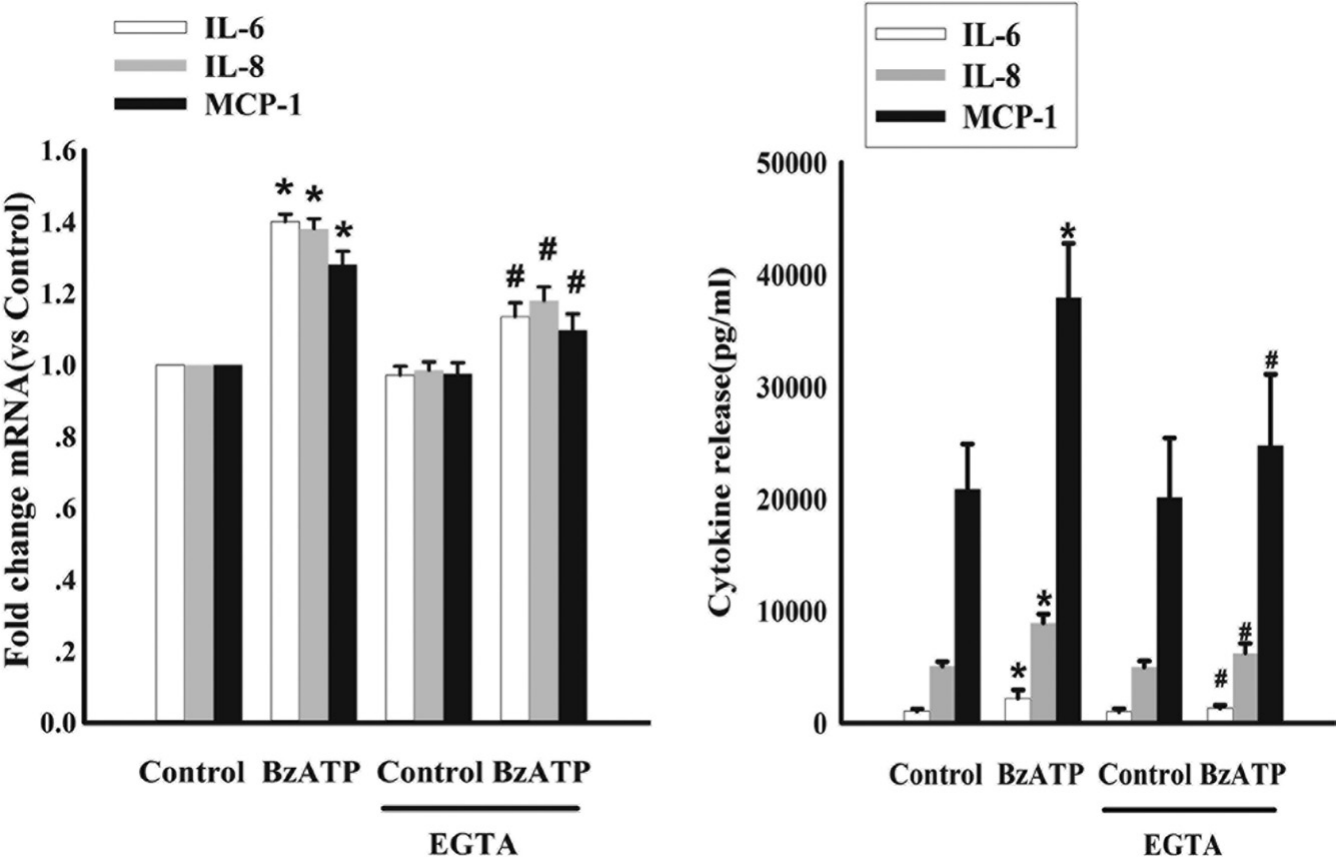
P2X7R modulates IL-6, IL-8 and MCP-1 expression and release through P2X7R-induced Ca^2+^-dependent signaling. Cells were pretreated with the extracellular Ca^2+^ chelator EGTA (1 mM) for 30 min and then exposed to 300 μM BzATP for 48 h. The relative fold change of expression of the IL-6, IL-8 and MCP-1 was determined by RT-PCR (left). Cytokine release levels in primary cultured CP cells were measured using a Millipore multiplex bead array in a Luminex 100 system (right). Data are representative of three independent experiments; bars represent the means ± SEM from triplicates. Statistical significance was determined by ANOVA analysis with *post hoc* Tukey’s test. *P < 0.05 vs respective control Group; #*P* < 0.05 vs the cultures exposed to BzATP (300 μM) alone.

It is known that BzATP induces Ca^2+^ influx through P2X7R (Shieh *et al*. 2014). Hence, we investigated whether Ca^2+^ played a role in the P2X7R-mediated increased expression of IL-6, IL-8 and MCP-1. To this end, we pretreated cultured CP cells with 1 mM of EGTA for 30 min and then exposed them to BzATP for 48 h. As shown in Fig. 5, the expression and release of IL-6, IL-8 and MCP-1 in BzATP-treated cells were attenuated in the presence of EGTA compared with those seen in BzATP-treated controls. This suggested that the P2X7R-mediated increase in IL-6, IL-8 and MCP-1 involves a Ca^2+^-dependent mechanism.

## Discussion

Craniopharyngiomas, though histologically benign and amenable to surgical resection, are nevertheless associated with a high degree of morbidity mostly because of their capacity to invade the surrounding structures. Here, we find that tumor tissue is marked by elevated levels of pro-inflammatory cytokines. It is likely that these cytokines are synthesized by infiltrating leukocytes but based on the analysis of isolated tumor cells *in vitro*, they may also be generated and released by tumor cells themselves. Nevertheless, it remains to be established whether the elevated plasma levels of the respective cytokines that we observed in CP patients are entirely due to the local production within and around the tumor.

Numerous studies of a variety of tumors show that local inflammation promotes angiogenesis, tumor growth, invasion and metastasis (Wculek & Malanchi 2015, Komohara *et al*. 2016). Nevertheless, the notion that local inflammation might be responsible for the invasive growth of CP is so far based on correlations. Interestingly, the cytokines we found to be expressed at highest levels, IL-6, IL-8 and MCP-1, have been previously shown to be involved in local inflammation and promotion of tumor growth including invasion and metastasis (Conti & Rollins 2004, de la Iglesia *et al*. 2008, Grivennikov & Karin 2008, Chalaris *et al*. 2011). It is hence conceivable that these three cytokines, and likely others we have not specifically analyzed, are at least in part responsible for the capacity of CP cells to invade and damage the surrounding tissue. We know that IL-1beta can be a potent tumor promoter (as well as a key pro-inflammatory cytokine), and our data show that the cultured CP cells could release IL-1beta, but the concentration of IL-1beta released from cultured CP cells is very low. The data give us the information that the source of IL-1beta in CP tissue may be produced by both infiltrating inflammatory cells and CP cells themselves.

Human craniopharyngioma consists of two histologically, genetically and clinically different entities (adamantinomatous and papillary type), and 48 patients with a postoperative pathological diagnosis of CP were studied in our paper. Our data show that of the 10 cytokines tested, MCP-1 and TNF-α were significantly upregulated in aCP (adamantinomatous type) patients compared to pCP (papillary type) patients. There is no difference between pediatric and adult CP patients, and there is also no difference between pediatric and adult aCP patients (as showed in Supplementary Fig. 1, see section on supplementary data given at the end of this article).

If indeed cytokine accumulation were responsible for CP invasion, and CP without invasion were more readily treatable by surgery, then understanding the mechanisms of cytokine induction might lead to the design of rational approaches to more successful therapeutic interventions. It is in this context that we focused on the role of the purinergic P2 receptor P2X7R. This ion channel can be stimulated by ATP, which is present in abundance around CP tumors. Indeed, we found that P2X7R is expressed in CP tumors *in vivo* both by RT-PCR and immunohistochemistry, and in primary cultured tumor cells by RT-PCR, Western blot and immunofluorescence. We further found that treatment of tumor cells with siRNAs against P2X7R reduces, and treatment with a potent receptor agonist, BzATP, stimulates the production and release of IL-6, IL-8 and MCP-1. This stimulation involved a Ca^2+^-dependent mechanism as would be expected if it were directly related to P2X7R activation. Hence, we conclude that high levels of ATP are at least in part responsible for the local cytokine induction around CP tumors. Of course, the reason for the elevated levels of ATP still needs to be investigated.

In summary, we demonstrated that P2X7R is expressed in CP and that its activation controls synthesis and release of IL-6, IL-8 and MCP-1 in CP. This pathway may provide a strategy that CP cells employ to invade normal brain structures. As inhibition of P2X7R signaling can ameliorate ATP-mediated neuronal and glial excitotoxicity and experimental autoimmune encephalomyelitis (Matute *et al*. 2007, Sharp *et al*. 2008, Komohara *et al*. 2016), it may also become a therapeutic strategy in CP. Indeed, antagonists for P2X7R are currently being considered as candidates to control CP progression, mitigate the inflammatory response, reduce intraoperative damage to critical structures and thus reduce the risks associated with surgical resection.

## Supplementary Material

Supporting Figure 2

Supporting Figure 2

## Declaration of interest

The authors declare that there is no conflict of interest that could be perceived as prejudicing the impartiality of the research reported.

## Funding

This study was supported by key Clinical Specialty Discipline Construction Program, National Key Technology Support Program (2014BAbib4B01), President Foundation of Nanfang Hospital, Southern Medical University (2015bib28).
