# Supplementary figures and images for: The purine receptor P2X7R regulates the release of pro-inflammatory cytokines in human craniopharyngioma
Source: Endocr Relat Cancer. 2017 Apr 7;24(6):287–96. doi: 10.1530/ERC-16-0338 (PMC5457505; doi:10.1530/ERC-16-0338)

**A**

Plasma

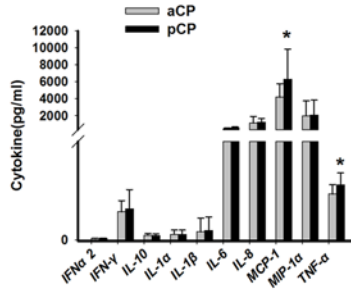**B**

Plasma

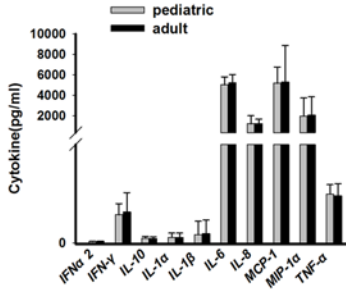**C**

Plasma

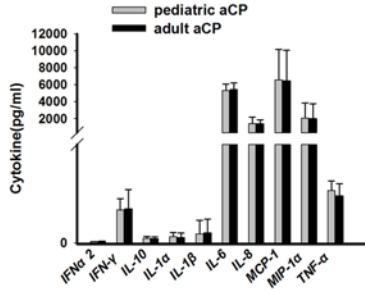

Supplement: Supporting Figure 2 [file erc-24-287-s001.pdf]

**A**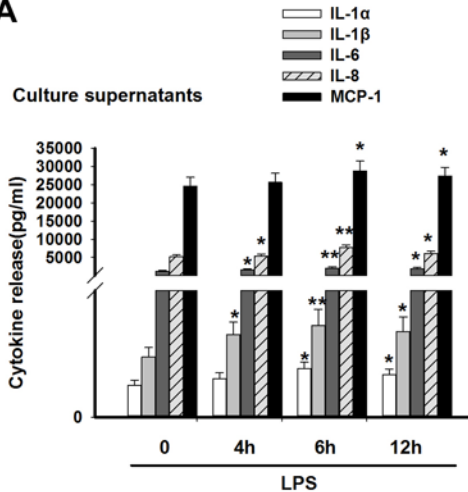**B**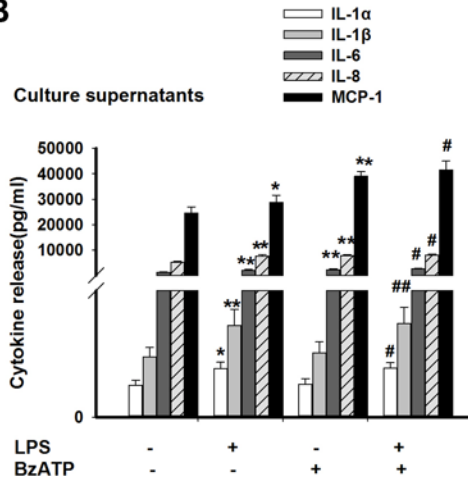

Supplement: Supporting Figure 2 [file erc-24-287-s002.pdf]
